# Psychological Theoretical Frameworks of Healthy and Sustainable Food Choices: A Systematic Review of the Literature

**DOI:** 10.3390/nu16213687

**Published:** 2024-10-29

**Authors:** Elena Lo Dato, Sara Gostoli, Elena Tomba

**Affiliations:** Department of Psychology, University of Bologna, 40126 Bologna, Italy; elena.lodato2@unibo.it (E.L.D.); sara.gostoli2@unibo.it (S.G.)

**Keywords:** sustainable eating, healthy eating, psychological theoretical framework

## Abstract

Background: Non-sustainable diets are associated with several environmental and health-related problems. Psychology research is interested in the study of food choice determinants, and several theoretical frameworks have been applied to study mechanisms underlying behavioral change and to develop theory-based interventions. The present systematic review is aimed at reviewing the existing literature on the psychological theoretical frameworks used to study sustainable and/or healthy food choices and their application for the development of interventions promoting such food choices, both in general and clinical populations. Methods: A systematic search of PubMed, PsycInfo, and Scopus was conducted according to PRISMA criteria. Results: Forty-five articles met the inclusion criteria and thirty-five theoretical frameworks emerged, mostly pertaining to social psychology and with the most widely used being the Theory of Planned Behavior. The majority of studies had a cross-sectional design, were conducted in general populations, and focused on healthy food choices. Only a few studies tested theory-based interventions. Internal (i.e., self-efficacy, personal values, and motivation) and external (i.e., peers, family, and social media influence) factors emerged as relevant healthy and sustainable eating determinants. Conclusions: The current review underlines that an integrative perspective combining prompts from different psychology fields is needed in order to identify the psychological factors influencing food choices and to develop psychological interventions for the promotion of more sustainable diets.

## 1. Introduction

Research on sustainable and healthy eating determinants and on how to encourage more sustainable diets is growing. Non-sustainable and unhealthy diets indeed have a huge impact both at the environmental level, contributing to land degradation, deforestation, loss of biodiversity, and the contamination of air, soil, and water [1], and at the individual level, leading to serious physical and mental health consequences [2]. The increase in the production of cheap, palatable, and energy-dense foods, which are made much more convenient and accessible by distribution systems and more appealing by food marketing, has also contributed to a substantial increase in obesity rates [3], with one in every three people worldwide affected by this condition [4]. The increase in obesity rates is alarming, considering not only its economic burden, accounting for 0.7% to 2.8% of a country’s total healthcare expenditure [5], but also its well-known association with non-communicable diseases (NCD) [6], low quality of life, and psychological problems [7,8,9]. At the same time, more sustainable food choices, such as reducing meat consumption, have been found to be associated with higher levels of psychological well-being [10] and lower levels of stress, depression, and anxiety, as assessed by the Depression and Anxiety Stress Scale (DASS-21) [11].

In line with the One Health Approach, an integrated and unifying approach aimed at balancing and optimizing the health of people, animals, and the environment, and that mobilizes different sectors and disciplines to create long-term and sustainable solutions to global health threats [12], research from different disciplines has increasingly focused on the study of healthy and sustainable food choice determinants [13,14,15] to create a One Health network addressing the challenges of shifting eating habits towards healthier and more sustainable diets [16].

Although many individuals might want to change their behaviors towards more sustainable ones, behavioral change is not always easy to reach, and translating intention into action can be challenging. In psychology, so-called health behavior change research is interested in the study of the processes and mechanisms underlying health behavioral change, with the ultimate goal of aiding individuals in engaging in healthy lifestyles and promoting their well-being [17]. Several models and theories have been developed as the groundwork for understanding behavioral change mechanisms and developing effective interventions, given that theory-based interventions have been shown to be more effective than non-theory-based ones [18]. However, to date, most of these theories and models have been applied to the study of limited types of health behaviors, such as physical activity and healthy nutrition, whereas only a few studies have focused on the domain of sustainable eating.

The aim of the present study was to systematically review the existing literature on the psychological theoretical frameworks used to study sustainable and/or healthy food choices and their application for the development of interventions promoting such food choices, both in general and clinical populations. Providing an extensive overview of the psychological theoretical frameworks applied to study healthy and sustainable food choices may indeed be useful both for researchers and clinicians to identify psychological factors influencing food choices, as well as to identify and develop effective psychological interventions to tackle these psychological features for the promotion of healthier and more sustainable diets.

## 2. Methods

### 2.1. Search Strategy and Eligibility Criteria

The present systematic review was conducted according to the Preferred Reporting Items for Systematic Reviews and Meta-Analyses (PRISMA) [19] guidelines, with the aim of reviewing the existing literature on psychological theoretical models applied to the study of sustainable and/or healthy food choices. The systematic search was conducted between January and May 2024 on Pubmed, PsychInfo, and Scopus, combining the following keywords: psychological theory OR psychological model OR psychological framework AND healthy diet* OR healthy nutrition OR healthy food choice* OR sustainable food choice* OR sustainable eating. The search strategy was restricted to titles and abstracts. Studies were also identified through citation searching (references lists). Filters were applied to the search concerning language (only articles in English) and the type of article, while the search was not limited by year of publication. In particular, empirical articles published in peer-reviewed academic journals were included in the research, while case studies, study protocols, books and chapters of books, other systematic and non-systematic reviews, and meta-analyses were filtered out from the research. Furthermore, studies were included in the review if they presented at least one psychological theoretical model applied to the study of sustainable and/or healthy food choices, excluding general food choices, and were conducted in general and/or clinical populations.

### 2.2. Selection Process

Titles and abstracts of selected studies were first screened by one author (E.L.D.) with the support of a master’s student in clinical psychology. Articles that appeared potentially relevant for the purpose of the review were independently reviewed and assessed by the other two authors (E.T., S.G.), who found consensus for eligibility. In cases of disagreement, multiple rounds of full-text revisions and discussions were held until agreement was reached by all three authors.

Data were extracted from the articles included in the systematic review by the three authors. Inclusion and exclusion criteria and data extraction were based on patient, intervention, comparison, outcome, and study design (PICOS) criteria when applicable. Please see Table 1 for further details on the criteria used for data extraction.

## 3. Results

### 3.1. Study Selection

**Figure 1:**
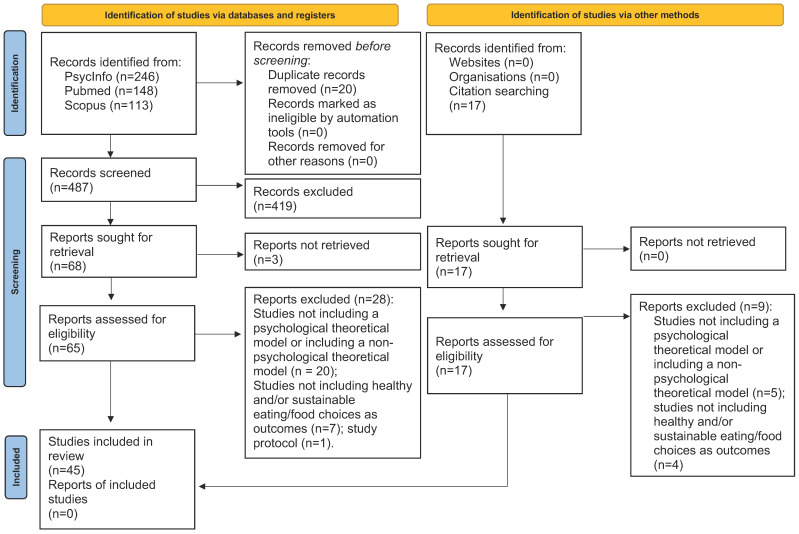
PRISMA 2020 flow diagram for new systematic reviews that included searches of databases, registers, and other sources.

The literature search yielded a total of 507 records, of which 246 were from PsycInfo, 148 from Pubmed, and 113 from Scopus. After duplicate removal, a total of 487 records were screened. The first screening of titles and abstracts led to the exclusion of 419 articles, mainly because of not including a psychological theoretical model or including a theoretical model pertaining to other disciplines (i.e., marketing, economy, food science). Sixty-eight articles were sought for retrieval, of which three were not retrieved. Thus, sixty-five articles underwent full-text reading, leading to the exclusion of a further twenty-eight articles. An additional seventeen records were identified through citation searching, of which nine were excluded. Therefore, a final set of 45 articles identified through databases and citation searching was included in the present review. See Figure 1 for more details on the study selection process.

### 3.2. Study Characteristics

Of the 45 included studies, 23 had a cross-sectional design [20,21,22,23,24,25,26,27,28,29,30,31,32,33,34,35,36,37,38,39,40,41,42], 9 a longitudinal design [43,44,45,46,47,48,49,50,51], 12 an experimental design [35,36,52,53,54,55,56,57,58,59,60,61]—of which 8 were randomized controlled trials (RCT) [36,52,53,55,56,57,59,62], 4 were laboratory studies [35,58,60,61] and 1 was an uncontrolled clinical trial [63]—and 2 had a quasi-experimental design [40,64]. Note that three of the studies [35,36,40] comprised two sub-studies with different research designs.

Healthy food choices represented the main outcome variable in most of the included studies (86.7%), whereas only six studies [22,24,37,41,42,62] explicitly took into account sustainable eating. Furthermore, while the majority of studies (88.9%) were conducted with participants from the general population, five focused on clinical populations, including women attending primary health care services [57], individuals at risk of coronary artery disease [44], patients in rehabilitation for cardiac and orthopedic problems [46], patients with a metabolic syndrome [31], and overweight or obese college students [27]. See Table S1 in the Supplementary Materials for further details on study characteristics.

### 3.3. Psychological Theoretical Frameworks Applied to Study Healthy and/or Sustainable Food Choices

Within the 45 included studies, a total of 35 different theoretical frameworks emerged, including both original versions of existing theories and extended versions or combinations of two or more theories. A more detailed description of the original versions of the psychological theoretical frameworks is presented in Table S2 in the Supplementary Materials. The majority of the identified theoretical frameworks pertained to the field of social psychology (N = 15; 42.8%), with the most widely used theory being the Theory of Planned Behavior (TPB) [65], followed by cognitive psychology (N = 11; 31.4%), health psychology (N = 6; 17.1%), clinical psychology (N = 3; 8.6%), humanistic psychology (N = 2; 5.7%), cross-cultural psychology (N = 2; 5.7%), and developmental psychology (N = 1; 2.8%) (note that some models resulted from the integration of two or more theories pertaining to different psychology fields).

A synthesis of the studies’ relevant findings, organized according to the applied theoretical frameworks pertaining to each of the aforementioned psychological fields, will be presented in the following sections. Please see Table S1 in the Supplementary Materials for further details on study characteristics.

### 3.4. Social Psychology

#### 3.4.1. Theory of Planned Behavior (TPB)

Among the social psychology theoretical frameworks, the TPB—in its original formulation or in extended versions of the theory, including additional antecedents of the intention and/or the behavior—was the most widely used [23,27,32,34,36,38,40,41,55,59,64].

Three studies applied the TPB in their original formulations in relation to healthy food choices in the general population [32,40,64], showing that attitude and perceived behavioral control (PBC) were the most significant predictors of the intention to eat healthily in dyads of mothers and children from the UK [32] and that all TPB predictors equally predicted healthy food choices in Iranian healthy women [40]. Furthermore, TPB-based educational interventions showed promising results in terms of increasing fruit and vegetable consumption, subjective norms, PBC, attitude, and behavioral intentions towards fruit and vegetable consumption post-intervention in Iranian elementary students [64], and in terms of improving both the intention and the behavior of healthy eating in adults from the general population at the 3-month follow-up (FU) [40], as compared to the no-intervention control groups [40,64].

Most of the studies applying extended versions of the TPB (E-TPB) proposed and tested additional predictors of the intention to eat in a healthy and/or sustainable way, including personality dimensions [27], past behaviors [36], self-identity [34,36,38], anticipated regret [59], health value [34], and personal norms [38]. In terms of healthy eating, the predictive value of the original TPB predictors of intention was confirmed in samples of Italian [36], American [27], Australian [34], and Turkish [38] adults from the general population. Additional significant predictors of the intention included the personality dimension of consciousness in non-clinical young adults [27], personal norms in terms of commitment to internalized values and feeling of personal obligation to perform a certain behavior [38], and self-identity as a healthy eater in non-clinical adult populations [34,38]. The latter also emerged as a significant predictor of behavior in both adults from the general population and pregnant women [34,38]. Two E-TPB-based experimental interventions, consisting of filling in a food diary and receiving daily reminders about reducing red meat consumption (RMC) [36], as well as self-monitoring RMC and potential anticipated regret associated with RMC [59], were effective in increasing the intention to reduce RMC, promoting an actual reduction in RMC [36,59] and its anticipated regret [59] in non-clinical young adults compared to the control groups (i.e., food diary and self-monitoring only).

Three studies testing E-TPB proposed possible additional antecedents of the predictors of intention, including outcome evaluations [23], perceived facilitation [23], motivation [23,55], and normative, control, and behavioral beliefs [23,41]. In terms of healthy eating, gender differences emerged in samples of Latin American adolescents, with females’ attitudes being determined by outcome evaluations in terms of feeling healthy and looking good, and subjective norms being determined by their mothers’ influence, while males’ attitudes were determined by outcome evaluations in terms of having a good athletic performance [23]. Self-efficacy emerged as a significant predictor of the intention of eating healthier in a non-clinical adult Belgian population [55]. Furthermore, an E-TPB-based experimental intervention improved autonomous motivation, which in turn positively predicted changes in attitude, self-efficacy, and intention to eat healthy and to engage in physical activity in a sample of adults from the general population as compared to the usual care control condition [55]. Only one study took into account sustainable eating in terms of meat consumption, carrying out an assessment through an ad hoc self-report questionnaire in an adult non-clinical German population [41]. Attitude was mostly determined by behavioral beliefs in terms of outcome expectations, subjective norms were determined by normative beliefs in terms of the belief that a significant other expects us to perform a behavior, and PBC was determined by control beliefs in terms of the belief that factors facilitating or hindering a behavior (i.e., relevant resources, such as cooking skills) are relevant, and the intention to reduce meat consumption was significantly predicted by the original TPB variables [41]. See Table S1 for further details on study characteristics.

#### 3.4.2. Social Cognitive Theory (SCT)

Three studies [20,21,28] applied the SCT [66] regarding healthy food choices. Self-efficacy significantly predicted healthy eating in American children [20], while self-efficacy together with self-regulatory behaviors, social support, and negative outcome expectations in terms of physical (i.e., immediate sensory experiences such as taste), social (i.e., devoting too much time and energy to nutritional goals), and self-evaluative (i.e., emotional response to change) expectations significantly predicted healthy eating in American adults [21]. In addition, one study showed that mothers of overweight and obese children in China had lower self-efficacy in ensuring daily fruit and vegetable consumption in their children [28] compared to mothers of non-obese children. Intervention studies applying the SCT did not emerge from the present review. See Table S1 for further details on study characteristics.

#### 3.4.3. Influence of Presumed Media Influence (IPMI)

In line with the IPMI theory [67], one study [30] found that perceived media influence on others indirectly influenced intentions to engage in healthy eating through attitudes and personal norms in a sample of adults from the Japanese general population [30]. Intervention studies applying the IPMI theory did not emerge from the present review. See Table S1 for further details on study characteristics.

#### 3.4.4. Information–Motivation–Behavioral Skills (IMB) Model

One study [31] applied the IMB model [68] in a sample of patients with metabolic diseases (Mets) from South Korea, showing that healthy eating behaviors were directly influenced by behavioral skills (i.e., being able to exercise regularly) and motivation, which in turn was influenced by information on Mets. In addition, distress levels indirectly influenced healthy behaviors through motivation and behavioral skills [31]. Intervention studies applying the IMB model did not emerge from the present review. See Table S1 for further details on study characteristics.

### 3.5. Cognitive Psychology

#### 3.5.1. Dual Process Theory (DPT)

The DPT [69] was applied in two studies to explore individuals’ decision-making processes in terms of healthy food choices [29,35]. In line with the theory, adults from the Dutch general population with higher levels of hunger had lower levels of self-control toward healthy food choices, even though hunger did not directly influence food choices [35]. In a subsequent experimental study, hungry participants made less healthy food choices in the food choice task than satiated participants, but only in the absence of a social proof heuristic (i.e., seeing that the majority of previous participants had chosen the healthy food option), whereas the social proof heuristic led hungry participants to make as many healthy food choices as satiated participants did [35]. In terms of DPT-based interventions, one study showed that the application of interventions targeting automatic decision-making processes (i.e., nudging-like interventions—NLI) was positively valued by a non-clinical Danish sample of adolescents, with the strongest positive effect on participants’ attitudes toward NLI being their sense of responsibility and their healthy buffet habits (i.e., the tendency to select healthy food options, for example vegetables, at a buffet) [29]. See Table S1 for further details on study characteristics.

#### 3.5.2. Muddling-Through Theory (MTT)

A theoretical framework based upon the MTT [70], advocating that attitudes towards giving up unhealthy food choices and choosing healthy foods were predicted by COVID-19 fear and health consciousness, which in turn were related to the social influence of family, peers, and the media, was developed and tested in a non-clinical adult population from Turkey [39]. While family and social media influence had a positive effect both on COVID-19 fear and health consciousness, peers had a positive influence only on health consciousness. In addition, both health consciousness and COVID-19 fear positively affected attitudes towards healthy eating, which in turn was positively associated with healthy nutrition. Intervention studies applying the muddling-through theory did not emerge from the present review. See Table S1 for further details on study characteristics.

#### 3.5.3. Value–Attitude–Behavior (VAB) Model

Based on the Value–Attitude–Behavior model (VAB) [71], an E-VAB was developed and tested in a sample of adults from the American general population when dining out [25]. Health value represented the main predictor of healthy food choices, hedonic and positive outcome expectations, and interest in healthy food, with customers’ intentions to eat healthy food being enhanced by hedonic positive outcome expectations [25]. Intervention studies applying the VAB model did not emerge from the present review. See Table S1 for further details on study characteristics.

#### 3.5.4. Habit-Formation Theory (HFT)

In line with the HFT [72], enhanced intrinsic reward levels, anticipated regret, and self-efficacy were associated with better overall automaticity of healthy food choices within the same day in a non-clinical sample of German adults in relation to healthy eating [51]. Intervention studies applying the HFT did not emerge from the present review. See Table S1 for further details on study characteristics.

#### 3.5.5. Implicit Misattribution Model (IMM)

The IMM [73] was applied in one study with young adults from the American general population [58]. In the food domain, the IMM consists of pairing (i.e., presenting paired stimuli in word format) different food stimuli in order to promote behavioral change towards healthier food choices. In the included study, positively conditioned healthy food stimuli (CS+, i.e., grapefruit) were paired with positive unconditioned stimuli (US+, i.e., fit), while negatively conditioned unhealthy food stimuli (CS-, i.e., cheeseburger) were paired with negative unconditioned stimuli (US-, i.e., bulky). The eating intentions of participants assigned to an experimental condition (CS+ and US+ pairing) were found to be more sensitive to food health than food taste as compared to the control group (no CS-US pairing) [58]. The finding was confirmed by a subsequent study, but only when participants were asked to categorize foods based on mealtime (i.e., breakfast vs. dinner) and on their level of healthiness (i.e., healthy vs. unhealthy) [58]. Intervention studies applying the IMM did not emerge from the present review. See Table S1 for further details on study characteristics.

#### 3.5.6. Associative–Cybernetic Model

The associative–cybernetic model [74] was applied in one study with reference to healthy food choices in a non-clinical adult population from Germany [45]. In terms of healthy eating, the intrinsic reward value of fruit and vegetable consumption had an indirect positive effect on habit strength through its influence on fruit and vegetable consumption frequency. Moreover, the relationship between habit strength and fruit and vegetable consumption frequency was moderated by rewarding levels; in other words, consumption frequency had a stronger effect on habit when consumption was perceived as more rewarding [45]. Intervention studies applying the associative–cybernetic model did not emerge from the present review. See Table S1 for further details on study characteristics.

#### 3.5.7. Cross-Behavior Regulation

In line with the cross-behavior regulation framework [24], regular exercise predicted healthier diets six months later in German patients in cardiac and orthopedic rehabilitation, and patients who engaged in regular exercise at baseline also reported higher levels of exercise strength six months later [46]. Furthermore, patients with greater habit strength were more likely to make use of transfer cognitions, meaning that their acquired competencies in the exercise domain also facilitated future learning in the nutrition domain [46]. Intervention studies applying the cross-behavior regulation model did not emerge from the present review. See Table S1 for further details on study characteristics.

#### 3.5.8. Social Learning Theory (SLT)

In line with the SLT [75], Austrian children exposed to an audio-visual cartoon in a “minority” condition (i.e., watching a single child eating a healthy food) made less healthy food choices than children in the “majority” condition (i.e., watching a group of children eating a healthy food) and in the control condition (i.e., no food placement), confirming the influence of peers in children’s food choices [60]. Intervention studies applying the SLT did not emerge from the present review. See Table S1 for further details on study characteristics.

#### 3.5.9. Prospect Theory

In line with the prospect theory [76], Austrian children in a gain-frame condition (receiving messages on the advantages of eating fruit), as compared to peers in a loss-frame condition (receiving messages on the disadvantages of not eating fruit) and a no-frame condition (no messages), showed healthier food choices in a snack choice test (fruit vs. candies) [60]. Intervention studies applying the prospect theory did not emerge from the present review. See Table S1 for further details on study characteristics.

#### 3.5.10. Self-Licensing Theory

In line with the self-licensing theory [77], Swiss adult participants recalling a past completed egoistic action were more likely to make healthy food choices and were willing to pay more for healthy products as compared to those recalling a past completed altruistic action (study 1), whereas participants that intended to perform an altruistic action were more likely to choose healthy food as compared to those who intended to perform an egoistic action (study 2) [56]. Intervention studies applying the self-licensing theory did not emerge from the present review. See Table S1 for further details on study characteristics.

### 3.6. Health Psychology

#### 3.6.1. Health Action Process Approach (HAPA)

The HAPA [78] was used in four studies in relation to healthy food choices [26,33,47,48]. Outcome expectancies and perceived self-efficacy predicted healthy eating intentions in terms of low-fat and high-fiber diets, and intentions predicted behavior in a sample of adults from the German general population [47]. In a sample of young adults from China, the relation between the intention to eat healthy in terms of fruit and vegetable consumption and the behavior itself was mediated by action control and action planning [48]. An E-HAPA model including the role of transfer cognitions and compensatory health beliefs (CHBs; i.e., beliefs that an unhealthy behavior, such as unhealthy eating, may be compensated for by engaging in another healthy behavior, such as physical activity) was developed [26]. In line with the theory, transfer cognitions were significantly associated with intentions to eat healthy and with self-regulatory strategies [26], while CHBs were only associated with intentions. Similarly, CHBs were significantly and negatively associated with intentions and action planning in non-clinical adult populations from Germany, Italy, Greece, and Spain when trying to reduce unhealthy eating [33]. Intervention studies applying the HAPA did not emerge from the present review. See Table S1 for further details on study characteristics.

#### 3.6.2. Health Belief Model (HBM)

Two studies [43,52] applied the HBM [79]. Based on an E-HBM, it was found that all E-HBM variables significantly predicted sodium consumption, with affect, perceived consequences, and personal norms predicting behavioral intention and habit predicting meat consumption in a sample of university students in the USA [43]. In another study, an HBM-based intervention was effective in increasing nutritional knowledge and decreasing fat and cholesterol consumption, as compared to a no-treatment control group, in a sample of adults from the American general population [52]. See Table S1 for further details on study characteristics.

#### 3.6.3. Temporal Self-Regulation Theory (TST)

In line with the TST [80], the intention to consume fruits and vegetables in a sample of students from the UK was predicted by their beliefs about the short- and the long-term positive outcomes; beliefs of short-term negative outcomes were negatively associated with fruit and vegetable consumption intentions. Furthermore, fruit and vegetable consumption were predicted by intentions and past behaviors, whereas unhealthy snacking was predicted by habit strength and past behavior [50]. Intervention studies applying the TST did not emerge from the present review. See Table S1 for further details on study characteristics.

### 3.7. Clinical Psychology

#### 3.7.1. Mindfulness Theory

Based on the mindfulness approach [81], three of the included studies applied and tested mindfulness-based interventions for the promotion of healthy [63] and sustainable eating in terms of organic, local, and seasonal food purchase and reduced meat consumption, assessed through an ad hoc self-report questionnaire [37] and in terms of ecological and economic impact through the Sustainable Consumption Behavior–Nutrition Scale [62] in the German general population. In terms of healthy eating, 1 h mindfulness group training was effective in promoting healthier food choices in American students post-intervention [63]. In terms of sustainable eating, sustainable food consumption was significantly and directly associated with the mindfulness dimension “acting with awareness”. In addition, it was indirectly associated with the mindfulness dimension “observing” through construction of meaning, sustainability-related beliefs of meaning, and personal norms in a non-clinical sample of German adults [37]. Furthermore, a consumption-specific mindfulness-based intervention (MBI) focused on mindful eating and sustainable nutritional behavior was effective in enhancing mindful eating and strengthening pre-consumption intentions and attitudes, as compared to a waiting list control group, in a sample of German students [62]. See Table S1 for further details on study characteristics.

#### 3.7.2. Transtheoretical Model (TTM)

Two of the included studies applied the TTM [82] to the development of psychological interventions promoting healthy food choices [53,57]. In line with an E-TTM including, along with the TTM stages, TPB predictors of intention as predictors of stages of change (SOC), an attitude change intervention (i.e., providing a brief leaflet including messages designed to persuade people that eating a low-fat diet would help them maintain fitness and enhance feelings of health) advanced participants from the precontemplation to the contemplation stage, whereas a perceived behavioral control change intervention (i.e., providing low-fat options that participants could easily incorporate within their lifestyle) advanced participants from both the contemplation and action stages, as compared to an information-only control group in a sample of adults from the general population in the UK [53]. Another TTM-based psychological group intervention consisting of a pre-action (i.e., workshops on cognitive and experiential processes of change) and an action (i.e., workshops on behavioral processes of change, including practicing healthy food preparation) phase was effective in improving body perception and promoting weight loss and the lower consumption of caloric and high-in-fat foods in a sample of Brazilian women attending a primary health care facility, as compared to a routine-care control group [57]. See Table S1 for further details on study characteristics.

### 3.8. Humanistic Psychology

Self-Determination Theory

Three of the included studies applied the SDT [83] to study healthy [44,49] and sustainable [24] food choices in both clinical and general populations. One study showed that Canadian patients at risk of coronary artery disease who were more generally self-determined at baseline were more likely to regulate their eating behaviors for self-determined reasons at the 13-week FU, with a subsequent decrease in the consumption of unhealthy foods at the 26-week FU [44]. Sustainable eating in terms of meat and plant-based food consumption, assessed through an ad hoc self-report questionnaire, was taken into account in one study of the Dutch general population [24]. Internally motivated participants reported reduced meat consumption and were more likely to choose plant-based products compared to externally motivated participants [24]. Similarly, another study showed that autonomous forms of motivation predicted healthier food choices, while external motivation was predictive of unhealthy food choices in a sample of Swiss adults from the general population [49]. Intervention studies applying the SDT did not emerge from the present review. See Table S1 for further details on study characteristics.

### 3.9. Cross-Cultural Psychology

#### Theory of Basic Values

The theory of basic values [84] was used in two of the included studies in the context of sustainable food choices, in both cases in terms of meat consumption, and assessed through an ad hoc self-report questionnaire [22] and a food choice task [42]. Based on an extended theoretical framework combining the theory of basic values [84], the Dual-Process Theory [69], and the Regulatory Focus Theory [85,86], universalism (i.e., understanding, tolerating, and protecting the welfare of others and of nature) significantly predicted reduced meat or free-range meat consumption in a non-clinical sample of Dutch adults [22]. Conservation values (i.e., values that emphasize stability, preservation, and resistance to change) were negatively associated with sustainable food choices, while self-transcendence values (i.e., values that emphasize the welfare and interest of others, nature, and the environment) were positively associated with sustainable food choices both when eating at home and at restaurants, with health motives driving home meal choices and social and taste motives driving restaurant meal choices in Dutch adults from the general population [42]. Intervention studies applying the theory of basic values did not emerge from the present review. See Table S1 for further details on study characteristics.

### 3.10. Developmental Psychology

#### Piaget Theory

Only one study [54] applied a developmental psychology framework to study healthy food choices in children from Turkey. Based on Piaget’s cognitive development theory [87], a game-based nutritional educational intervention aimed at improving nutritional knowledge (i.e., on the food pyramid, food groups, and fruit and vegetable consumption) was developed and was found to be effective in increasing children’s nutritional knowledge and healthy food choices as compared to children participating in a general educational program [54]. See Table S1 for further details on study characteristics.

## 4. Discussion

The importance of healthy and sustainable eating, both for our own health and environmental welfare, has become a topic of global interest. Nevertheless, despite an awareness of the benefits that healthier and more sustainable diets might have for their health and for the environment, people find it difficult to change their eating habits due to deep-seated psychological reasons [88]. Achieving such a behavioral change is often challenging, and several psychological theories have been proposed to study this complex domain and to identify strategies to alter these behaviors. The aim of the present systematic review was to provide an overview of the psychological theoretical frameworks used to study sustainable and/or healthy food choices and their application in the development of interventions promoting sustainable and/or healthy food choices, both in general and clinical populations. Several theories from different psychological fields emerged, showing that behavioral change results from the interaction between multiple factors, and highlighting the need for an integrative perspective to study healthy and sustainable food choices, in line with the One Health Approach [12].

One key finding emerging from the present review is the limited psychological literature on sustainable eating. Indeed, only six of the included studies explicitly included sustainable eating as an outcome variable [22,24,37,41,42,62]. According to the Food and Agriculture Organization (FAO), sustainable diets have a multi-dimensional nature and are defined as “protective and respectful of biodiversity and ecosystems, culturally acceptable, accessible, economically fair and affordable; nutritional adequate, safe and healthy; while optimizing natural and human resources” [89]. In the reviewed studies, sustainable eating was mostly defined in terms of food choices only, and a shared conceptualization and operationalization of the construct of sustainable eating was lacking. Despite food choices representing a central feature of sustainable eating, other equally important dimensions not directly related to food choices, such as storing and preserving food (i.e., checking expiration dates of food products), cooking (i.e., cooking the right amount of food), food consumption (i.e., use of plastic cutlery), and food disposal (i.e., separating and recycling garbage) [90], were not taken into account in the included studies. In addition, sustainable eating was primarily assessed through ad hoc self-report questionnaires developed for specific studies; only one study [62] used an existing questionnaire, namely the Sustainable Consumption Behavior–Nutrition Scale, highlighting the need for a more comprehensive and multi-dimensional definition of sustainable eating, as well as the identification of integrative assessment tools able to capture all its dimensions.

Another notable finding is that only five studies were conducted in clinical populations [27,31,44,46,57]. Although studying healthy and/or sustainable food choices in the general population is crucial for the prevention of eating-related health problems, the impact of unhealthy and unsustainable eating behaviors in certain clinical populations cannot be overlooked. Despite evidence showing that the consumption of unhealthy and non-sustainable foods is largely responsible for the increase in obesity rates observed in recent decades [91], and that interventions promoting sustainable diets may have positive effects in overweight patients [92], only one study included in the present review involved patients with obesity [27]. Obesity is a stigmatized condition, and individuals with obesity are often described as not motivated, lazy, lacking in self-discipline, and thus responsible for their own weight and health condition [93,94]. Although the lack of studies on healthy and sustainable eating in this clinical population may in part be due to our limited search strategy, obesity stigma may also explain this result. Stigma could indeed also be shared among healthcare professionals, given that patients themselves sometimes describe physicians as one of the main sources of stigma [95,96].

Regarding the identified psychological theoretical frameworks and related theory-based interventions, social psychology theories were the most widely used, consistent with studies on behavioral change in other health behaviors [97,98]. In particular, the TPB emerged as the most frequently used one, with studies supporting its value in predicting the intention of engaging in healthy [27,32,34,36,38,40] and sustainable [41] eating behaviors. The TPB also emerged as a commonly used theoretical background for the development of interventions to promote healthy and/or sustainable food choices [36,40,55,59,64], and it has been found to be effective for the promotion of such behaviors. In line with this finding, the TPB has been widely used to develop behavioral change interventions (i.e., through goal-setting, planning, and self-monitoring) across a variety of health behaviors (i.e., alcohol consumption, nutrition, physical activity, sexual behavior, etc.), with significant effects, albeit varying based on the specific addressed health domain [99]. Cognitive psychology theories also emerged as common theoretical frameworks to study healthy [25,29,35,39,45,46,50,56,58,60,61] and sustainable [22] food choices, highlighting the importance of both conscious and unconscious cognitive mechanisms in the actualization of health behaviors and the achievement of behavioral change [100]. For instance, non-intrusive interventions targeting unconscious decision-making processes, such as nudging interventions, have been developed and widely applied with promising results for promoting healthy and sustainable food choices [101,102,103]. However, studies applying cognitive psychology theories in the present review were mostly theoretical.

Research in clinical psychology is also extending its focus to the study of healthy and sustainable food choices. Mindfulness-based theories emerged as frequently used theoretical frameworks to study healthy [63] and sustainable [37,62] food choices, as well as to develop theory-based interventions for the promotion of such behaviors, in line with recent findings supporting the use of the mindfulness approach to encourage other health-related behaviors, such as physical activity, healthy eating, and sleep [104]. The mechanisms underlying the improvement of the mentioned behaviors through mindfulness include enhancing self-regulation strategies, increasing knowledge of positive health practices, and boosting motivation [105], all features that emerged as crucial for healthy and sustainable eating behaviors in several theoretical frameworks analyzed in the present review [21,24,31,35]. In addition, mindful eating (i.e., a non-judgmental acceptance of physical and emotional states while eating) helps in the regulation of internal cues, such as hunger and satiety, to avoid the overconsumption of food driven by external stimuli [106] and can foster healthier food choices [107]. Despite mindfulness in general being deemed to be useful for the promotion of sustainable consumption [108,109], only one of the included studies tested a mindful eating approach for the promotion of more sustainable eating behaviors [62].

Within the clinical psychology field, a stage-based theory also emerged from the present review. The TTM has been applied in two intervention studies [53,57], in both cases supporting its effectiveness in advancing individuals from earlier stages of readiness to change (i.e., precontemplation) to later stages (i.e., action), supporting the notion that individuals at different stages of change might benefit from different intervention components and that the one-size-fit-all approach may be limiting [17].

Despite the vast heterogeneity of psychological theories emerging from the present review, some studies have integrated existing theoretical frameworks with additional variables or theories, revealing an overlap of some key constructs and bringing us closer to achieving an integrative and comprehensive perspective. The findings of the present review highlight how behavioral change should be seen in terms of a dynamic interplay between internal and external factors, in line with a previous systematic review on food choice determinants [110]. On the one hand, individual factors such as intention, perceived behavioral control, self-efficacy, motivation, values, self-regulation, and taste emerged as relevant food choice determinants across the life span and among different cultures, aside from the psychological field in which they were tested [20,21,31,35,41,47,49,51,55]. Within this integrative perspective, eating does not represent merely a biological need and it is not always driven by a physical state of hunger, but it is also connected with the pleasure deriving from food consumption, lifestyle choices, and one’s own values. In this sense, psychological interventions aimed at boosting individuals’ confidence in their ability to make healthier food choices and at enhancing self-efficacy may be useful to promote healthier dietary habits [111]. For example, a recent study [112] testing the effects of text-message-based interventions that involved the reading of either pre-factual well-being/health messages (i.e., “If you follow a diet with plenty of animal proteins and fat, this will have a negative impact on your well-being/health”) or factual well-being/health messages (i.e., “Following a diet with plenty of animal proteins and fats has a negative impact on your well-being/health”) showed that eating self-efficacy moderates the effects of well-being/health messages on red meat consumption. Specifically, factual health messages persuaded participants with high self-efficacy, while pre-factual well-being messages persuaded participants with average levels of self-efficacy. The mentioned study provides interesting insights, on the one hand, on the role of persuasive communication to promote healthier food choices and, on the other hand, on the role that one’s own self-efficacy may have in changing eating habits, further supporting the need for a multi-dimensional and integrative perspective. In clinical contexts, a shared decision-making approach, consisting of a joint decision-making process between healthcare professionals and patients to reach health-related decisions, may be particularly useful in this regard. Patients may indeed have the opportunity to fully understand the risks and the benefits associated with a certain health-related decision and to make informed choices on their health [113].

On the other hand, social and interpersonal factors, such as the influence of social media, peers and family, social support, and social norms, were also found to play a role and to influence food choices [21,30,39,60], highlighting the need to also consider the influence of external factors for the promotion of more sustainable eating behaviors. In addition, receiving information on healthy and/or sustainable diets also seems to play an important role in behavioral change [31,52,54]. This finding aligns with several studies showing that health literacy, defined as individuals’ knowledge, motivation, and competences to access, understand, and use health information to make health-related decisions [114], is associated with several health behaviors [115,116,117,118], and it is often used as a strategy to promote such behaviors [119,120,121].

Overall, the findings of the present review may be useful to foster better eating habits that contribute both to individual health and to the achievement of broader sustainability goals. On the one hand, healthcare professionals, including dieticians, psychologists, and nutritionists, should consider the role that societal and interpersonal factors, such as families and partners, may have in the management of eating-related conditions (i.e., obesity), along with individual characteristics. Indeed, it has been highlighted that family lifestyle and eating habits strongly influence family members’ food choices [122]. On the other hand, since providing a growing global population with more sustainable habits represents one of the main challenges of recent decades [6], the findings of the present review may inform future policies and guidelines for the promotion of more sustainable and healthier diets at the community level.

## 5. Limitations and Future Directions

The results of the present review should be considered in light of its limitations. First of all, due the descriptive nature of this systematic review, primarily aimed at providing an overview of the existing psychological theoretical models of sustainable and/or healthy eating behaviors, we did not provide a novel/advanced psychological theoretical model of healthy and sustainable food choices. With regard to search strategies, only three databases were used for study selection, and the grey literature was not taken into account, which might have led to the exclusion of relevant studies not retrieved through the consulted databases. The combination of keywords referring to psychological theoretical frameworks in general (i.e., “psychological theory”, “psychological model”, “psychological framework”) might also have led to the exclusion of relevant studies. However, considering that the aim of the present review was to review the existing literature on psychological theoretical frameworks pertaining to different psychological fields, this search strategy might be considered as the most appropriate for our purpose.

Despite these limitations, the present systematic review offers important insights on the study of healthy and sustainable food choices from a psychological point of view, which should be considered for future studies. An integrative perspective integrating prompts deriving from different psychology fields, such as social, cognitive, clinical, and cross-cultural psychology, is needed in order to fully understand psychological determinants of healthy and sustainable food choices. Furthermore, studies should focus more on psychological determinants of sustainable food choices both in general and clinical populations. In order to do so, a comprehensive and shared definition of the construct is needed, as well as the use of multi-dimensional measures for its assessment. In addition, a greater focus should be put on the identification of psychological interventions aimed at promoting more sustainable food choices in general populations. At the same time, psychological interventions aimed at reducing non-sustainable eating habits in clinical populations, such as patients with obesity, diabetes, and cardiovascular diseases, should also be tested.

## 6. Conclusions

In recent years, psychology research has extended its focus to the study of sustainable eating in order to shed some light on the role that psychological factors may have in the choice of healthy and sustainable food products. However, the study of healthy and sustainable food choices has interested a number of different psychology fields, spanning social, cognitive, and clinical psychology, making the identification of psychological factors affecting food choices oftentimes challenging. The present review provides an overview of the most widely used psychological theoretical frameworks applied to study healthy and sustainable food choices and their application for the development of theory-based psychological interventions to promote healthier and more sustainable diets both in general and clinical populations, offering useful insights to researchers and clinicians. It may indeed aid in the identification of existing theoretical frameworks applied to the study of healthy and sustainable eating behaviors, as well as in developing and testing extended versions of these theories in order to increase their predictive power. In addition, it may be useful for the identification of psychological interventions for the promotion of healthy and sustainable food choices both in general and clinical populations.

## Figures and Tables

**Table 1:** PICOS criteria for data extraction.

PICOS	Inclusion Criteria	Exclusion Criteria	Data Extraction
Population	All ages All genders Both general and clinical populations		Number of participants Gender Mean age Diagnosis (when applicable)
Intervention	All types of psychological interventions		Treatment characteristics when provided
Comparison group	Studies with and without comparison groups		Control group characteristics when applicable
Outcome	Psychological theoretical frameworks to study healthy and/or sustainable food choices and their application to the development of psychological interventions promoting such diets	Studies taking into account general food choices without referring to healthy and/or sustainable eating specifically or proposing theoretical frameworks pertaining to other disciplines (i.e., marketing, economy, food science)	Differences between participants or modifications of outcome variables over time
Study design	Empirical studies (cross-sectional, longitudinal, RCT, quasi-experimental)	Reviews Summaries Essays Panel studies Theoretical proposals	Study design Study setting

## Supplementary Materials

The following supporting information can be downloaded at: https://www.mdpi.com/article/10.3390/nu16213687/s1, Table S1: Study characteristics; Table S2: Description of the identified psychological theoretical models.
