# Supplementary material for: Psychological Theoretical Frameworks of Healthy and Sustainable Food Choices: A Systematic Review of the Literature
Source: Nutrients. 2024 Oct 29;16(21):3687. doi: 10.3390/nu16213687 (PMC11547315; doi:10.3390/nu16213687)
Supplement: Supplementary file 1 [file nutrients-16-03687-s001.zip › nutrients-3254684-supplementary.pdf]

Table S1. Study characteristics.

Authors	Country	Study design	Psychological theoretical model	Sample characteristics	Eating outcome	Measures/tasks	Intervention	Relevant findings
**Social psychology**								
Sumodhee & Payne, 2016 [32]	UK	Cross-sectional	Theory of Planned Behavior (TPB)	60 dyads of mothers and their children (n=60 mothers aged 54±4.25; n=60 children aged 24±4.35, 65% females)	Healthy eating	*Eating Attitude Test* (EAT-26) to assess disordered eating behaviors; *Ad-hoc self-report* TPB-based questionnaire to assess beliefs about healthy eating; *Child Feeding Questionnaire* (CFQ) to assess parental feeding practices	Not applicable	Maternal restrictive feeding practices were negatively associated with their own attitude and subjective norms (SN) and with their adult children' SN. Furthermore, mothers' TPB beliefs and intentions were positively associated with children' TPB beliefs and intentions. Both for mothers and children, attitude was the strongest predictor of intention, followed by perceived behavioral control (PBC). Finally, mothers' PBC and intention significantly predicted their children intention to eat healthy food
Taghdisi et al., 2016 [64]	Iran	Quasi-experimental (pre/post-test)	TPB	184 healthy children (100% males; mean age not reported)	Healthy eating	*Self-report ad-hoc questionnaire* to assess TPB variables; *24-h recall questionnaire* to assess FV consumption	Experimental group: four 45-min training sessions for students and 60-min training sessions for parents and teachers to increase SN about FV consumption of students. In the first	Whereas no significant differences were observed in both predictors of the intention and FV consumption between groups at baseline, an increase in FV consumption and in the mean scores of the predictors of the intention were observed in the experimental group as

							and second training sessions, students were given information on types of food groups and units of FV consumption; the third session was aimed at improving the attitude towards FV consumption and the PBC and at identifying barriers of FV consumption and strategies to overcome them.<br><br>Control group: no intervention	compared to the control group following the intervention
Jeihooni et al., 2021 [40]	Iran	Cross-sectional (study 1); Quasi-experimental (study 2; pre/post/3-months FU)	TPB	Study 1: 350 healthy adults (age: 39.58 ± 4.86; 100% women).<br><br>Study 2: 200 healthy adults (experimental group: n=100; age: 38.80 ± 4.75; 100% women; control group: n=100; age: 39.25 ± 4.20; 100% women)	Healthy eating	*Self-report ad-hoc questionnaire* to assess TPB variables and nutritional behaviors	Experimental group: eight 50-min group sessions on health education and promotion of healthy diets preventive of cardiovascular diseases<br><br>Control group: no intervention	Attitudes, SN and PBC significantly predicted healthy nutritional behaviors (study 1). The TPB-educational intervention was effective in promoting healthy nutritional behaviors as compared to the control group, but only at 3-months FU (study 2)

Monds et al., 2016 [27]	Australia and USA	Cross-sectional	E-TPB including the HEXACO – humility-emotionality-extraversion-agreeableness-conscientiousness-openess – personality dimensions as additional predictors of the intention	1036 healthy young adults (age: 23.08 ± 7.43; 63.9% females) classified as underweight (n=41), healthy weight (n=501) and overweight or obese (n=475)	Healthy eating	*Self-report ad-hoc questionnaire* to assess TPB variables and FV consumption; *International Personality Item Pool* to assess the HEXACO domains of personality	Not applicable	Overall, the E-TPB model was predictive of FV consumption and the personality dimension consciousness was a significant predictor of the intention. In the healthy weight group only, PBC significantly predicted intention, which in turn was predictive of the behavior
Malek et al., 2017 [34]	Australia	Cross-sectional	E-TPB including self-identity as a healthy eater, health value and perceived stress as additional predictors of the intention	455 pregnant women (age: 31.6 ± 4.9)	Healthy eating	*Self-report ad-hoc questionnaire* to assess TPB original and additional variables, dietary intake and adherence to food group recommendations (based on Australian Dietary Guidelines Five Food Group System)	Not applicable	PBC and SN were the most significant predictors of the intention, followed by attitudes and self-identity. Self-identity as a healthy eater also emerged as a significant predictor of the behavior
Carfora et al., 2017a [36]	Italy and UK	Cross-sectional (study 1) and RCT (study	E-TPB including past behaviors and self-identity in terms of healthy-eating identity and meat-eating identity as	Study 1: 342 healthy young adults (age: 19.58±2.03; 71% females)	Healthy eating	*Self-report ad-hoc questionnaire* to assess TPB original and additional variables; Food diary to monitor food	Experimental group: participants received a daily message reminding their goal to reduce RMC (not exceeding two portions per week)	Study 1 showed that the strongest predictors of the intention to reduce RMC were PBC and affective attitude, while healthy eating identity did not represent a significant predictor.

		2; pre/post-test)	additional predictors of the intention	Study 2: 228 healthy young adults assigned to the experimental group (n=116; age: 19.29±1.75; 72% females) or the control group (n=112; age: 19.29 ± 1.04; 71% females)		consumption and healthy eating in terms of red meat consumption (RMC)	and filled in a daily food diary<br><br>Control group: participants filled in the daily food diary only	In study 2, participants in the experimental group, compared to the control group, increased their intention to reduce RMC and reduced their consumption. Sequential mediation analyses also showed that the increase in the healthy eating identity was associated with a reduction in their meat-eating identity, increasing intention and therefore reducing RMC at T2 in the experimental group
Carfora et al., 2017b [59]	Italy and UK	RCT (pre/post-test)	E-TPB including anticipated regret as additional predictor of the intention	112 healthy young adults (age: 19.37 ± 1.55; 56% females; n=55 experimental group; n=57 control group)	Healthy eating	*Self-report ad-hoc questionnaire* to assess TPB original and additional variables;<br>Food diary to monitor food consumption and healthy eating in terms of RMC	Experimental group: participants were asked to self-monitor their RMC daily and received a daily message focused on anticipated regret and a reminder to monitor RMC<br><br>Control group: participants were only self-monitoring their RMC	Participants in the experimental group significantly increased their intention to reduce RMC, reduced their RMC, and increased anticipated regret following intervention compared to the control group. Furthermore, the effectiveness of messages compared to no messages was mediated by sequential effects of messages on anticipated regret and intention
Ates, 2019 [38]	Turkey	Cross-sectional	E-TPB including self-identity and personal norms as antecedents of both the intention and the behavior	279 healthy adults (age: 35.4; 65% females)	Healthy eating	*Self-report ad-hoc questionnaire* to assess TPB original and additional variables and healthy nutritional behaviors	Not applicable	Overall, the E-TPB model significantly predicted both the intention and the behavior, with personal norms, self-identity and PBC being the most significant predictors of the intention and self-identity

								and PBC being the most significant predictors of healthy nutritional behaviors
Diaz et al., 2009 [23]	USA	Cross-sectional	E-TPB including behavioral beliefs and outcome evaluations as antecedents of the attitude, normative beliefs and motivation to comply as antecedents of SN and control beliefs and perceived facilitations as antecedents of PBC	265 healthy adolescents (56% females; mean age not reported)	Healthy eating	*Self-report ad-hoc questionnaire* to assess the TPB variables; *Short acculturation scale for Hispanics (SASH)* to assess the acculturation variables	Not applicable	Females showed stronger intention, more positive attitudes and greater subjective norms influences compared to males. Feeling healthy and looking good for females and having a good athletic performance for males significantly predicted attitudes, while the influence of mothers significantly predicted subjective norms in females. Furthermore, receiving support and encouragement represented a significant predictor of PBC in less acculturated adolescents
Seffen & Dohle, 2023 [41]	Germany	Cross-sectional	E-TPB including beliefs as antecedents of the intention and past behavior and habit strength as additional predictors of both the intention and the behaviors	1093 healthy adults (age: 44.35 ±14.51; 50.3% males)	Healthy and sustainable eating	*Self-report ad-hoc questionnaire* to assess TPB variables, behavioral, normative and control beliefs, past behavior and general intention of meat consumption; *Self-Report Behavioral Automaticity Index*	Not applicable	The E-TPB overall predicted and explained the motivation to reduce meat consumption, with SN and PBC significantly predicting the intention. Adding past behavior and habit as predictors of the intention did not improve the model's predictive power. Attitude was predicted by behavioral beliefs (i.e., eat a healthier diet), SN by normative beliefs (i.e., family

						to assess habit strength		expectations) and PBC by control beliefs (i.e., time).
Sharma et al., 2006 [20]	USA	Cross-sectional	Social Cognitive Theory (SCT)	159 healthy children (age: 69.9% 10 y/o; 45.6% males)	Healthy eating	*Self-report ad-hoc questionnaire* to assess the four outcome behaviors (FV consumption, PA, watching TV and water consumption) and related outcome expectations and self-efficacy	Not applicable	Self-efficacy of eating FV significantly predicted FV consumption
Anderson et al., 2007 [21]	USA	Cross-sectional	SCT	712 healthy adults (age: 53.54±14.37; 66% females)	Healthy eating	*Food Belief Survey* to assess social cognitive variables (social support, self-efficacy, outcome expectations and self-regulation); *Self-report ad-hoc questionnaire* based on family food shopping and the FFQ to assess nutrition behaviors	Not applicable	Self-efficacy, enacted self-regulatory behaviors, social support and negative outcome expectations significantly predicted healthy eating behaviors
Xu et al., 2016 [28]	China	Cross-sectional	SCT	1208 mothers of preschool children (n=120 mothers of overweight and	Healthy eating	*Self-report ad-hoc questionnaire* to assess maternal cognitions on their	Not applicable	Positive correlations were observed between maternal social cognitions and PA, but not with healthy nutrition

				obese children, age: 30.79±3.66; n=966 mothers of normal weight children, age=31.87±4.19; n=122 mothers of underweight children, age=31.94±3.45)		child PA and healthy nutrition behaviors		behaviors. Mothers of obese and overweight children had less self-efficacy in ensuring their children to eat FV everyday
Ho et al., 2016 [30]	Japan	Cross-sectional	Influence of presumed media influence (IPMI) model (extended version including attitude, injunctive and personal norms on individuals' intention to engage in physical activity and a healthy diet)	1055 healthy adults (age: 42.99±14.51; 57.25% females)	Healthy eating	*Ad-hoc self-report questionnaire* to assess attention to media, perceived influence, attitudes toward a healthy lifestyle, injunctive and personal norms and healthy lifestyle behavioral intention	Not applicable	Perceived media influence indirectly influenced intentions to engage in a healthy life-style through personal norms and attitudes
Lee et al., 2016 [31]	South Korea	Cross-sectional	Information-motivation-behavioral (IMB) skills model	267 patients with metabolic syndromes (Mets) (age: 54±8.1; 54.3% women)	Healthy eating	*Ad-hoc self-report questionnaire* to assess information (knowledge and recognition of healthy behaviors for Mets), motivation toward a healthy lifestyle, behavioral skills	Not applicable	Personal and social motivation and behavioral skills directly influenced healthy behaviors in patients with Mets, whereas psychological distress indirectly influenced healthy behaviors through motivation and behavioral skills

						and health behavior;<br>the *Brief Encounter Psychological Instrument* to assess psychological distress		
**Social and humanistic psychology**								
Jacobs et al., 2011<br>[55]	Belgium and UK	RCT (pre/post-test)	E-TPB including the Self-Determination Theory (SDT) variables autonomous and controlled motivations as antecedents of the predictors of the intention (attitude and self-efficacy)	287 healthy adults (age: 40.48 ± 10.55; 66.5% males)	Healthy eating	*Self-report ad-hoc questionnaire* to assess TPB and SDT variables; items selected from the *Behavioral Regulation Exercise Questionnaire II* and the *Treatment Self-Regulation Questionnaire* to assess autonomous and controlled motivations of physical activity and dietary behaviors	Experimental group: educational website and one-to-one or group coaching sessions in addition to usual care to improve attitudes, self-efficacy and motivation toward physical activity and dietary behaviors. Participants could freely decide the intervention intensity (frequency)<br><br>Control group: usual care (i.e, medical screening)	Changes in autonomous motivation significantly predicted changes in attitudes, self-efficacy and intentions to eat healthy.<br>Self-efficacy significantly predicted intention in both contexts (PA and dietary behaviors), whereas intervention intensity was a significant predictor of dietary behaviors change only
**Cognitive psychology**								
Nørnberg et al., 2016<br>[29]	Denmark	Cross-sectional	Dual-Process Theory	408 healthy adolescents (78.4% females, age:17.96; 21.6% males, age:18)	Healthy eating	*Self-report ad-hoc questionnaire* to assess FV consumption and the attitude toward school's rules and	Not applicable	The variables responsibility and healthy buffet habits (i.e., choosing healthy options at buffets) had the strongest positive effect on participants' attitudes toward NLI. Overall

						obligations in promoting healthy food choices as well as toward specific type of interventions (i.e., nudge-like interventions, NLI)		students were positive toward less intrusive interventions, such as those that rely on automatic decision-making paths instead of the reflective ones
Cheung et al., 2017 [35]	Netherlands	Study 1: cross-sectional Study 2: experimental (laboratory)	Dual-Process Theory	Study 1: 201 healthy adults (age: 37.67±12.72; 52.73% females)<br><br>Study 2: 188 healthy young adults (age: 20.66±2.47; 100% females)	Healthy eating	Study 1: *State Self-Control Capacity Scale* to assess their current state/mood<br><br>Study 2: *Product choice task:* participants were asked to choose between a total of ten food pairs, of which seven presented a self-control conflict (healthy vs. unhealthy food)	(Only for study 2) Social proof condition: during the product choice task, a social proof heuristic always promoted healthy food choices (i.e., participants were showed that the majority of previous participants chose the healthy option);<br><br>No heuristic condition: the product pairs were presented without extra information	In study 1, participants' self-reported hunger was negatively associated with self-control, but there were no effects of hunger on food choices. However, in study 2, hungry participants significantly made less healthy food choices than satiated participants when no social proof heuristic was present, while participants in the social proof heuristic condition made healthier food choices
Aksoy et al., 2021 [39]	Turkey	Cross-sectional	Muddling through theory	688 healthy adults (18.1% aged 18-25, 47.2% aged 26-39, 28.1% aged 40-54 and 6.6 % aged > 54; 68.1% females)	Healthy eating	*Ad-hoc self-report questionnaire* to assess family, peer and social media influence, fear of Covid-19, attitudes toward giving up	Not applicable	Family and social media influence significantly predictred Covid-19 fear and health consciousness, whereas peer influence significantly predicted health consciousness. Furthermore, both health

						unhealthy food choices and health consciousness		consciousness and Covid-19 fear positively affected attitude, which in turn was positively related to healthy nutrition
Kang et al., 2015 [25]	USA	Cross-sectional	E-Value-Attitude-Behavior (E-VAB) model including hedonic and positive outcomes expectations toward healthy food consumption, interest in healthy food consumption, health values and behavioral intention	1188 healthy adults (age: 53.2% between 18-34; 68.8% females)	Healthy eating	*Ad-hoc self-report questionnaire* to assess health value, positive outcome expectations, hedonic expectations, interest in healthy food and healthy food choices intentions	Not applicable	Health value was the main predictor of healthy food choices at restaurants, as well as a key predictor of hedonic expectations toward healthy food (both directly and indirectly through interest in healthy food), positive outcome expectations and interest in healthy food. Furthermore, customers' intentions to eat healthy were enhanced by hedonic expectations and positive outcome expectations
Di Maio et al., 2022 [51]	Germany, Poland and Australia	Longitudinal (12 weeks FU)	Habit formation theory	135 healthy young adults (age: 24.8±7.3)	Healthy eating	*Ad-hoc self-report questionnaire* to assess novel healthy eating behaviors-related automaticity, intrinsic rewards, anticipated regret and self-efficacy	Not applicable	Higher-than-usual levels of intrinsic reward, anticipated regret and self-efficacy of the same day but not of the previous day were associated with higher within-level automaticity
Bui & Fazio, 2016 [58]	USA	Experimental (laboratory)	Implicit misattribution model (IMM)	Study 1: 168 healthy young adults (58.3% females; mean age not reported).	Healthy eating	*Video Surveillance Procedure:* participants were asked to be vigilant for two target foods	Study 1 and study 2: Evaluative conditioning (EC) condition: CS and US were paired during the	Study 1: participants in the EC condition were less sensitive to food taste and more sensitive to food health in their eating intentions, showing that

				Study 2: 92 healthy young adults (32.6% females; mean age not reported)		(chicken pot pie vs. crackers) responding by hitting the space bar. Key conditioned stimuli (CS) and unconditioned stimuli (US) were embedded in this visual stream. CS were foods either high in taste and low in health or low in taste and high in health. Four healthy CS foods were paired with positive US, and four unhealthy CS foods were paired with negative US.<br><br>Following the task, participants were asked to rate the likelihood that they would eat an offered serving of 42 food items (including the eight CS foods seen in the previous task and 34 novel foods)	video surveillance procedure<br>Control condition: CS and US were presented separately<br><br>Study 2: participants were first asked to categorize foods based on meal-time (i.e., breakfast vs. dinner) and healthiness (healthy food vs. unhealthy food) and then completed the same task of study 1	conditioning a few exemplar food items increased sensitivity to health and decreased sensitivity to taste with a generalization effect of the association between healthy food and CS+.<br><br>Study 2: the generalization effect with regard to health sensitivity was confirmed, but only in participants who had categorized food by health

Wiedemann et al., 2014 [45]	Germany and UK	Longitudinal (2-weeks FU, T1, and 4-weeks FU, T2)	Associative Cybernetic Model	127 healthy adults (age: 31.7±10.1; 74% women)	Healthy eating	*Ad-hoc self-report questionnaire* to assess intention, self-efficacy (at baseline), FV consumption and intrinsic reward (at T1) and habit strength (at T2)	Not applicable	The intrinsic reward value of FV consumption strengthened FV consumption through two pathways: (1) intrinsic reward had an indirect effect on habit strength, whereby rewards influenced the frequency of eating FV, which in turn influenced habits; (2) moderating effect in which consumption frequency had a stronger effect on habit when consumption was deemed as more rewarding
Binder et al., 2019 [60]	Austria	Experimental (laboratory)	Social Learning Theory (SLT)	108 children (age=8.19±1.12; 59.3% females) assigned to the majority (n=39), minority (n=37) or control (n=32) condition and 108 parents (age=41.88±5.88; 77.1% women)	Healthy eating	*Snack choice test* to assess food choices (fruit vs. candy); *Food Neophobia Scale* to assess levels of neophobia of children from parents' perspective<br><br>*Task:*<br>children were exposed to an audio-visual cartoon movie in which only one child was eating raspberries (minority condition), a group of children were	Not applicable	Children in the minority condition, compared children in the majority and control conditions, were more likely to think that most of their peers did not like raspberries, leading to a lower likelihood to choose raspberries

						eating raspberries (majority condition) or none of the presented characters was eating raspberries or any other snack (control condition)		
Binder et al., 2020 [61]	Germany	Experimental (laboratory)	Prospect theory and the Reactivity of Embedded Food Cues in Advertising Model	161 children (age: 7.96±1.32; 47.2% girls) assigned to the control (n=48), gain-framed (n=55) or loss-framed (n=58) condition	Healthy eating	*Snack choice test* to assess food choices (fruit vs. candy); *Ad-hoc self-report questionnaire* administered to parents to assess nutritional mediation styles (restrictive or active)<br><br>*Task:* children were exposed to an audio-visual cartoon movie with gain-framed nutritional messages (experimental condition 1), loss-framed nutritional messages (experimental condition 2) or a message without	Not applicable	Overall, the study showed that framing nutritional messages in cartoon movies can positively influence children's fruit intake. Specifically, the gain-framed messages generated greater awareness of gain-framed arguments than the loss-framed and the control conditions. Even though both experimental conditions were effective in promoting fruit consumption, it seems to be easier for children to state gain arguments compared to loss arguments

						reference to food (control condition)		
Weibel et al., 2014 [56]	Switzerland	Experimental	Self-licensing and goal theory	Study 1: 62 healthy young adults (age: 23.98±3.51; 51.6% females)<br><br>Study 2: 106 healthy young adults (age: 23.61±8.14; 41.5% females)	Healthy eating	Study 1 and study 2:<br>*Task:* in a pre-test, participants evaluated 42 food options with regard to their healthiness and were then asked to choose four times between a healthy and an unhealthy food option;<br>*Willingness to pay* (WTP) to assess participants subjective value attributed to a certain product	Study 1:<br>Altruism condition: participants were instructed to recall an altruistic action carried out in the past and to write a short essay about it.<br>Egoism condition: participants were instructed to recall an egoistic action carried out in the past and to write a short essay about it.<br><br>Study 2:<br>Completed altruism: participants were instructed to recall a completed altruistic action.<br>Intended altruism: participants were instructed to recall an intended altruistic action.<br>Completed egoism: participants were instructed to recall a	Study 1: participants in the egoism condition chose healthy food options more often than participants in the altruism condition, supporting the idea that the activation of an altruistic or egoistic self-concept may influence healthy food choices. Furthermore, participants who wrote about an egoistic action were willing to pay more for healthy products than those who wrote about an altruistic action.<br>Study 2: action stage moderated the self-licensing effect on food choices. The intention to perform an altruistic action led participants to choose healthy foods more often, as well as to pay more for the products

							completed egoistic action.<br>Intended egoism: participants were instructed to recall an intended egoistic action	
**Cognitive and social psychology**								
Fleig et al., 2014 [46]	Germany	Longitudinal (6-months FU)	Cross-behavior regulation framework	470 patients in cardiac and orthopaedic rehabilitation (age: 50.46±9.07; 59% women)	Healthy eating	*Godin Leisure-Time Exercise Questionnaire* to assess physical exercise;<br>*Self-Report Habit Index* to assess exercise habit strength;<br>*Trasnfer Cognition Scale (TRACS)* to assess transfer cognitions at T2;<br>*ad-hoc self-report questionnaire* to assess healthy nutrition.	Not applicable	Regular exercise predicted engagement in healthy diets six months later. Furthermore, participants with more resources (i.e., higher levels of habit strength) were more likely to use transfer cognitions
**Health psychology**								
Schwarzer & Renner, 2000 [47]	Germany	Longitudinal (6-months FU)	Health Action Process Approach (HAPA)	580 healthy adults (age=43; 48% males)	Healthy eating	*Ad-hoc self-report questionnaire* to assess risk perception, outcome expectancies, action self-efficacy,	Not applicable	Overall, the more self-efficacious participants were, better nutrition behaviors were reported<br>Specifically, intentions were predicted by outcome expectancies and perceived

						intention, coping self-efficacy, low-fat dietary intake and high-fiber dietary intake		self-efficacy, with intentions representing good predictors of both low-fat and high-fiber dietary intake
Zhou et al., 2015 [48]	China, Germany, Australia and Poland	Longitudinal (2-weeks, T2, 4-weeks, T3)	HAPA	286 healthy young adults (age: 23.64 ± 4.44; 72.4% females)	Healthy eating	*Self-report ad-hoc questionnaire* to assess FV consumption intention and FV intake (at baseline), action control and action planning (at T2) and FV consumption (at T3).	Not applicable	Action control and action planning sequentially mediated the relation between intention to eat FV and future FV intake
Fleig et al., 2015 [26]	Germany, Canada, Spain, Greece, Italy and Australia	Cross-sectional	E-HAPA including cross-behavior cognitions	416 healthy adults from Southern Europe (age: 34.6) and 351 from Germany (age: 42.3)	Healthy eating	*General Practice Physical Activity Questionnaire* (GPPAQ) to assess physical acitivity; *The Mediterranean Diet Adherence Screener* to assess the adherence to a healthy diet; *Ad-hoc self-report questionnaire* to assess single health behavior cognitions (risk perception, positive outcome expectancies, self-efficacy, intentions, action planning and	Not applicable	Transfer cognitions were positively associated with intention to engage in PA and to eat healthy. Specifically, participants who believed that they could compensate for their unhealthy eating with regular exercise, reported lower healthy eating intentions. Contrarely, transfer cognitions were positively associated with healthy eating intentions, with participants believing that regular exercise supported their engagement in healthy dietary behaviors being more intended to stick to a healthy diet. Furthermore, action planning and action control mediated the

						action control) and cross-behavior cognitions (compensatory health beliefs and transfer cognitions)		relation between intention and action.<br>Finally, individuals with higher positive outcome expectancies and self-efficacy reported higher intention to engage in PA and consume a healthy diet
Amrein et al., 2017 [33]	Switzerland, UK and USA	Cross-sectional	E-HAPA including compensatory health beliefs	232 healthy adults (age: 27.3±8.5; 76.3% females) included for quantitative analyses; 79 healthy adults (age 26.26±8.84 for the FV consumption group and 28.09±10.05 for the unhealthy snack group; 68.4% females) included for qualitative analyses	Healthy eating	*Ad-hoc self-report questionnaire* to assess compensatory health beliefs (CHB), risk perception, self-efficacy, outcome expectancies, intention, action planning, action control and eating behaviors	As part of a previous RCT, participants were assigned to four experimental conditions in a 2x2 research design: different eating goals (increasing FV consumption or eating fewer unhealthy snacks) vs. intervention conditions (social support or control condition)	Quantitative findings showed that within the HAPA framework CHBs significantly predicted the intention but not the behavior and were significantly and negatively associated with intention and action planning only in the unhealthy snack condition, but not in the FV condition. Qualitative findings showed that CHBs were more likely to be present and were more diverse in the unhealthy snack condition. Overall, these findings suggest that CHBs play a more relevant role in relation to unhealthy snacking than fruit and vegetable consumption
Evans et al., 2017 [50]	UK	Longitudinal (1-week FU)	Temporal Self-Regulation Theory (TST)	133 healthy young adults (age: 23.92±7.40; 68.4% females)	Healthy eating	*Self-report ad-hoc questionnaire* to assess beliefs concerning FV consumption, connectedness	Not applicable	Beliefs about short-term and long-term positive outcomes predicted the intention to eat healthy, with intentions and past behavior predicting FV consumption. Furthemore, past

						beliefs, valence beliefs, timing beliefs; *Composite brief measure* to assess intentions, behavioral prepotency (past behavior frequency, habit strengths, perceived cues) and self-regulatory capacity		behavior moderated the relation between intention and behavior, which was stronger when past behavior was increasing

Abood et al., 2003 [52]	USA	RCT (pre/post-test)	HBM	63 healthy adults randomized to the experimental group (n=38; age=34.3; 96% females) or the control group (n=25; age=37.9; 92% females)	Healthy eating	*Ad-hoc self-report questionnaire* to assess HBM constructs (health concerns, perceived susceptibility, perceived severity, perceived benefits, perceived barriers and self-efficacy) in relation to nutrition, cardiovascular disease (CVD) and cancer; personal health assessment of risk of CVD and cancer; nutrition knowledge; dietary behaviors.	Experimental group: participants took part to eight 1-hour weekly educational sessions to promote knowledge and beliefs to improve or maintain positive dietary practices preventive of CVD and cancer.<br><br>Control group: participants only filled-in the baseline and post-intervention questionnaires	The HBM-based intervention was effective in producing significant increases in nutrition knowledge and significant decreases in energy, fat, saturated fat and cholesterol intake.
**Health and social psychology**								
Feldman & Mayhew, 1984 [43]	USA	Longitudinal (2-weeks FU)	E-Health Belief Model (E-HBM) integrating Fishbein's attitude-behavior theory and	120 healthy young adults (51.6% females; mean age not reported)	Healthy eating	*Ad-hoc self-report questionnaire* to assess behavioral intention, facilitating conditions, habit, affect, perceived	Not applicable	Overall, the model predicted both nutritional behaviors (sodium and meat consumption). Specifically, meat consumption was best explained by habit, whereas sodium consumption was

			Triandis' theory of social behavior			consequences, subjective values, normative beliefs, motivation to comply, role belief, personal normative belief, self-concept and meat and sodium consumption		predicted by habit, facilitating conditions and behavioral intention.<br>Affect represented the best predictor of sodium consumption behavioral intention, whereas personal norms beliefs, perceived consequences and affect significantly predicted meat consumption
**Clinical psychology**								
Jacobs et al., 2013 [63]	USA	Uncontrolled clinical trial	Mindfulness theory	26 healthy young adults (age=21.35±4.76; 77% females)	Healthy eating	*Toronto Mindfulness Scale* (TMS) to asses state mindfulness; *Philadelphia Mindfulness Scale* to assess present-moment awareness; *Five-Facet Mindfulness Scale* to assess mealtime awareness; *Ad-hoc self-report questionnaire* to assess healthy eating	1-hour mindfulness group training including didactic and experiential components	Following intervention, a significant increase in state mindfulness and in the rate of healthy consumption was observed, providing preliminary support that a brief session of mindfulness training can have an immediate effect in changing habitual responses to oneself and the environment
Hunecke & Richter, 2018 [37]	Germany	Cross-sectional (pre/post-test)	Mindfulness theory	310 healthy adults (age=36.1±11.8; 77% females)	Sustainable eating	*Ad-hoc self-report questionnaire* to assess personal norms, sustainable food consumption	Not applicable	The mindfulness dimension *acting with awareness* was significantly and directly associated with sustainable food consumption, whereas the

						behaviors (in terms of organic, local and seasonal food purchase and less meat food consumption), construction of meaning in life and sustainability-related meaning; *Five-Facet Mindfulness Questionnaire* to assess mindfulness		association between the dimension *observing* and sustainable food consumption was mediated by construction of meaning, sustainability-related meaning and personal norms
Stanszus et al., 2019 [62]	Germany	RCT (pre/post-test/7-months FU)	Mindfulness theory	76 healthy adults (age: 31; 71.34% women) assigned to the intervention group (n=37) or the control group (n=39)	Sustainable eating	*Comprehensive Inventory of Mindfulness Experience* (CHIME) to assess general mindfulness; *Mindful eating questionnaire* to assess mindful eating; *Ad-hoc questionnaire* to assess attitudes toward sustainable and healthy food consumption; *Sustainable Consumption Behavior-Nutrition scale* to assess	Experimental group: mindfulness-based intervention (MBI) consisting of eight weekly group sessions of 90 mintues and individual daily practices consisting of meditation practices, group discussions and guided reflections.<br><br>Control group: waiting condition.	MBI was effective in enhancing mindful eating. Effects on sustainable food consumption only appeared in the qualitative data as content related to pre-behavioral stages, attitudes and intentions. First FU results instead suggested a slower process for behavioral change toward more sustainable food choices

						sustainable eating behaviors (in terms of both ecological and economic impacts); *Qualitative interviews* to assess participants' general experience of the Mindfulness-based intervention (MBI), as well as their food behaviors and shopping routines and possible changes over those routines during the past weeks		

Menezes et al., 2015 [57]	Brazil	RCT (pre/post-test)	Transtheoretical model (TTM)	77 women attending the Primary Health Care assigned to the experimental group (n=43; age: 55.9 ± 9.7) or the control group (n=34; age: 60.4 ± 13.7)	Healthy eating	*Self-report ad-hoc questionnaire* based on the 24-h dietary recalls to assess food consumption and eating habits related to fat; *ad-hoc index* to assess stage of change (SOC)	Experimental group: routine activities (physical exercise, nutritional education and individual nutritional care) + SOC-specific interventions: pre-action group (participants in precontemplation, contemplation and preparation SOC) and action group (action and maintenance SOC).<br><br>Control group: routine activities (physical exercise, nutritional education and individual nutritional care)	Participants in the experimental group showed an improved body perception, reduced weight and BMI, and lower consumption of calories and foods high in fat post-intervention compared to the CG

**Clinical and social psychology**								
Armitage et al., 2004 [53]	UK	RCT (pre/post-test)	E-TTM including TPB variables as predictors of SOC	787 healthy adults (n=81 in the precontemplation stage, age=36.76 ± 10.38, 59.2% females; n=117 in the contemplation stage, age=34.17±11.15, 82.90% females; n=311 in the preparation stage, age=35.53±9.41, 84.88% females; n=79 in the action stage, age=38.54±11.01, 89.87% females; n=199 in the maintenance stage, age=37.61±9.87, 85.42% females)	Healthy eating	*Self-report ad-hoc questionnaire* to assess TPB variables and TTM stages of change towards healthy food choices.	Attitude change intervention: message designed to change the beliefs discriminating between individuals who were intended to eat a low-fat diet and those who were not intended to.<br><br>PBC change-intervention: enhancement of self-efficacy through the promotion of personal mastery and the use of persuasion, modelling and relaxation techniques.<br><br>Control group (information intervention): included current UK government recommendations concerning fat intake and a list of food high in fat.<br><br>All participants received a standard letter accompanying	The scores of the TPB increased in a linear fashion in successive SOC, with participants in later stages of change having more positive evaluations of eating a low-fat diet, perceiving more social pressure in doing so, being more confident in their ability to succeed and having a stronger intention compared to those in the earlier SOC. Furthermore, the attitude change intervention advanced individuals from the pre-contemplation stage, with age and PBC predicting progression from the contemplation and action stages; behavioral intention predicted progression from the preparation and maintenance stages and age predicted progression from the action stage

							their intervention leaflet and were randomized to two conditions:<br><br>Feedback condition: one additional sentence informing them on their current dietary fat intake<br><br>No feedback condition	
**Humanistic psychology**								
Pelletier et al., 2004 [44]	Canada	Longitudinal (13-weeks, T2, and 26-weeks, T3)	SDT	111 patients at risk for coronary artery diseases (age: 53.89±7.8; 40.5% females)	Healthy eating	*General Self-Determination Scale* to assess the general motivation (intrinsic, integrated, identified, introjected, extrinsic, amotivation); *Regulation of Eating Behaviors Scale* to assess the motivational orientation toward dietary regulation (contextual); *ad hoc* 24-h dietary recall questionnaire to assess food intake	Not applicable	General motivation was a significant predictor of contextual motivation, meaning that individuals who were more generally self-determined at baseline were also more likely to regulate their eating behaviors for self-determined reasons at T2. Self-determination at T2 was a significant predictor of the reduction of percent calories from total dietary fat and saturated fat at T3. Finally, the dietary behavior measures were related to improvements in weight and blood lipid parameters

Schösler et al., 2014 [24]	Netherlands and Germany	Cross-sectional	SDT	1083 healthy adults and young adults (age: 40% 35-54, 34% 55-74, 20% 18-34, 65% 75 and over; 50% females)	Sustainable eating	*Self-report ad-hoc questionnaire* to assess types of food-related motivations (intrinsic, extrinsic, internalized, not internalized), sustainable eating (in terms of meat consumption frequency, reasons for either a low or high meat consumption, purchase of organic meat, purchase of meat replacers, choices of plant-based snacks)	Not applicable	Internally motivated participants showed less meat consumption and preferred plant-based protein products compared to the externally motivated ones. Therefore, internalized motivation was the main factor determining sustainable food choices
Hartmann et al., 2015 [49]	Switzerland and Germany	Longitudinal (1-year FU)	SDT	2917 healthy adults (age: 58; 47% males)	Healthy eating	Adapted version of the *Treatment Self-Regulation Questionnaire (TSRQ)* to assess autonomous, introjected and external regulation towards a healthy body weight; *Food Frequency Questionnaire* to assess food and alcohol consumption;	Not applicable	Autonomous motivation predicted improvements in food choice and long-term PA, whereas external motivation predicted negative changes in food choices at the FU assessment

						Global Physical Activity Questionnaire-2 (GPAQv2) to assess PA		
**Cross-cultural psychology**								
Claessens et al., 2023 [42]	Netherlands	Cross-sectional	Theory of basic values	301 healthy adults (age: 51.4 ± 17.8; 49.2% females)	Healthy and sustainable eating	*10-Item Short Schwartz Value Survey* to assess participants' values; Adapted version of the *Food Choice Questionnaire* and the *Reasons to Snack Inventory* to assess food choices motives; *Hypothetical food choice task* to assess the healthiness and sustainability (in terms of meat consumption) of home and restaurants meal choices	Not applicable	Participants in the restaurant condition chose unhealthy meals more often than participants in the home condition. Furthermore, conservation values related negatively and self-trascendence values positively with sustainable food choices in both conditions. Regarding motives, taste and social eating were more important for choosing restaurant meals, whereas health was more important for choosing home meals. Finally, motives represented better predictors of healthy food choices in both conditions compared to sustainable food choices
**Cross-cultural and cognitive sychology**								

De Boer et al., 2007 [22]	Netherlands	Cross-sectional	Theory of basic values integrated with the Regulatory Focus Theory and the Dual Process Theory	1530 healthy adults and young adults (age: 18-89; 51% females)	Healthy and sustainable food choices	*40-item Portrait Value Questionnaire* to assess Schwartz basic values; *Ad-hoc self-report questionnaire* to assess sustaimable food choices and intentions (in terms of meat consumption)	Not applicable	Most of the basic human values were related to a certain extent to the direction of food choices motives. Universalism in particular significantly predicted less-meat or free-range meat food choices, with prevention-oriented food choice motives and high levels of food involvement and motive-congruent animal friendly attitudes mediating this relation
**Developmental Psychology**								
Baskale & Bahar, 2011 [54]	Turkey	RCT	Piaget's cognitive development theory	238 children (age: 5 y/o; 141 in the experimental group and 97 in the control group)ti	Healthy eating	*Ad-hoc self-report questionnaire* to assess children' nutritional knowledge, food consumption and anthropometric data	Experimental group: program of nutrition education based on Piaget's cognitive development theory constituted by an educational content and a game-based education.<br>Control group: general educational program.<br><br>Parents of children of both groups were given nutritional education in accordance with the	Children in the experimental group showed higher scores in nutritional knowledge compared to the control group and their food preferences positively changed toward healthier foods (i.e., white meat, fish, vegetables, fruits).

							principles of adult education	

Table S2. Description of the identified psychological theoretical frameworks.

**Psychological theoretical framework**	**Key factors**	**Description of the theory**
	**Social Psychology**	
Theory of Planned Behavior (TPB) [65]	• Intention<br>• Predictors of the intention (perceived behavioral control, PBC; social norms, SN; attitudes)	Behaviors are driven by individuals' intentions, which in turn are influenced by predictors of the intention (PBC, SN and attitudes).
Social Cognitive Theory (SCT) [66]	• Self-efficacy<br>• Outcome expectations<br>• Self-regulatory behaviors	Behaviors are influenced by levels of self-efficacy (individuals' ability to influence and change their habits) and physical, social and self-evaluative outcome expectations, both directly and through self-regulatory behaviors (in terms of setting, planning and monitoring personal goals).
Influence of Presumed Media Influence (IPMI) [67]	• Attitudes<br>• Media messages<br>• Social norms	Individuals adjust their attitudes and behaviors based on their perception of how others are influenced by media messages, that in turn are believed to indirectly influence behaviors through social

		norms and perceived influence on others.
Information-Motivation-Behavioral Skills (IMB) model [68]	• Information<br>• Motivation<br>• Behavioral skills	Information and motivation influence behavioral skills, which in turn determine behavioral change.
**Cognitive Psychology**		
Dual Process Theory (DPT) [69]	• Decision making processes	Two possible decision-making processes may drive behaviors: (1) a rational, deliberative, controlled and decision-making process; (2) an automatic, fast and habit-driven process.
Muddling Through Theory (MTT) [70]	• Decision making processes	In complex situations, decision-making driving behaviors may be based on a less rational approach, as more analytical and rational ones may be limiting.
Value-Attitude-Behavior (VAB) model [71]	• Abstract cognitions<br>• Mid-range cognitions	Behaviors are indirectly influenced by abstract cognitions (i.e., values) through mid-range cognitions (i.e., attitudes).
Habit Formation Theory (HFT) [72]	• Stages of behavioral adoption<br>• Intrinsic reward<br>• Anticipated regret<br>• Self-efficacy	Antecedents of behaviors can be distinguished based on the stage of behavioral adoption: (1) decision to act; (2) action initiation; (3) repetition of the behavior; (4) development of cue-behaviors association.<br>Within this habit formation process, antecedents such as intrinsic

		reward, anticipated regret and self-efficacy can play a role at multiple stages of the process.
Implicit Misattribution Model (IMM) [73]	• Evaluative conditioning	Behavioral change can occur when the value attributed to a certain stimulus (conditioned stimulus, CS) is paired with another positive or negative stimulus (unconditioned stimulus, US). Consequently, a CS may become more positive when paired with a positive US and more negative when associated with a negative US (evaluative conditioning).
Associative-cybernetic model [74]	• Reward value<br>• Behavior repetition<br>• Habit strength	Experiencing positive outcome behaviors when performing an action in a given context (i.e., enjoying the pleasurable taste of fruit when eating fruits at the dinner table in the evening) facilitates the learning of the behavior-context link.<br>The reward value (outcome) can strengthen habits via two routes: (1) reward should have an impact on habit that is mediated by behavior repetition; (2) reward should moderate the relationship between repetition and habit strength.
Cross-behavior regulation [26]	• Habit strength<br>• Transfer cognitions	Regular engagement in one health domain (i.e., physical exercise) can have implications for regulating

		other health domains (i.e., healthy eating), through habit strength and transfer cognitions.
Social Learning Theory (SLT) [75]	• Attention<br>• Retention<br>• Motor reproduction<br>• Motivation	The actualization of certain behaviors (i.e., food choices) may be influenced by others (i.e., peers).
Prospect Theory [76]	• Gain-framed messages<br>• Loss-framed messages	Gain-framed messages (i.e., advantages of eating fruit) more persuasively encourage preventive behaviors, while loss-framed ones (i.e., disadvantages of not eating fruit) more persuasively encourage behaviors aimed at detecting health conditions.
Self-licensing Theory [77]	• Moral self-concept<br>• Moral and immoral actions	Completed moral or altruistic actions boost people's moral self-concept, which decreases the tendency to act morally, while completed immoral or egoistic actions are compensated for by acting more morally or altruistically in the future.
**Health Psychology**		
Health Action Process Approach (HAPA) [78]	• Motivational phase of intention formation (self-efficacy, outcome expectancy and risk awareness)<br>• Volitional phase of translating the intention into	In the initial motivation phase, a person forms the intention to perform a certain behavior, which is predicted by self-efficacy, outcome expectations and risk awareness. Intention is then translated into action, which is influenced by action planning and action control.

	action (action planning and action control)	
Health Belief Model (HBM) [79]	• Health beliefs<br>• Readiness to take action<br>• Perceived susceptibility<br>• Severity<br>• Benefits<br>• Barriers	Health behaviors are determined by health beliefs and readiness to take action, which in turn are influenced by the levels of susceptibility, severity, benefits and barriers related to the specific health behavior.
Temporal self-regulation theory (TST) [80]	• Motivational phase (connectedness, timing and valence beliefs)<br>• Volitional phase (behavioral prepotency and self-regulatory capacity)	Health behaviors are driven by a motivational phase and a volitional phase. In the motivational phase, intention is thought to be influenced by beliefs about the connectedness (how likely an outcome of behavior is believed to be), valence beliefs (whether the outcomes are believed to be positive or negative) and timing beliefs (when the outcomes are believed to occur). In the volitional phase, intention is translated into action, which in turn is predicted by behavioral prepotency (the individual's default response to cues in the environment) and self-regulatory strategies (the individual's ability to monitor and control its thoughts emotions and behaviors).
**Clinical Psychology**		
Mindfulness theory [81]	• Observing<br>• Describing<br>• Acting with awareness	Behavioral change can result from the awareness that arises through paying attention on purpose in the

	• Non-judging<br>• Non-reactivity	present moment and with a non-judgemental attitude.
Transtheoretical model [82]	• Stages of change	Behavioral change crosses five stages, namely precontemplation, contemplation, preparation, action and maintenance, with transitions between stages being mostly predicted by decisional balance (i.e., valuing pros and cons of the behavior) and self-efficacy.
**Humanistic psychology**		
Self-determination theory (SDT) [83]	• Motivation<br>• Behavior regulatory styles	The regulation of behaviors can take many forms that correspond to different behavior regulatory styles and are associated with three forms of motivation, namely intrinsic motivation, extrinsic motivation and a-motivation.
**Cross-cultural psychology**		
Theory of basic values [84]	• Self-enhancement values<br>• Self-trascendence values<br>• Openness to change<br>• Conservation	Individuals' behaviors are influenced by values that are relevant for them. Values are organized on two bipolar dimensions: self-enhancement (concern for personal growth and success) vs. self-trascendence values (concern for the welfare and interest of others, the nature and the environment) and openness to change (independence of thoughts, action and feelings and readiness to

		change) vs. conservation values (preservation, stability and resistance to change).
**Developmental psychology**		
Piaget's cognitive developmental theory [87]	• Schemas<br>• Assimilation<br>• Accommodation<br>• Equilibration	Children's cognitive development is organized into stages based on their age and is based on their schemas. The progression of the development depends on its adaptation (including assimilation and accommodation), and as a result of the interaction between assimilation and accommodation, equilibration takes place, creating a new dynamic balance.
